# Guardians of T-Cell Ca^2+^ Stores: SERCA Pumps Integrated Within Complex Functional and Disease-State Signaling Dynamics

**DOI:** 10.3390/cells15131221

**Published:** 2026-07-06

**Authors:** MD Nasim Uddin, David W. Thomas

**Affiliations:** 1 Department of Cell and Biological Systems, Penn State College of Medicine, Hershey, PA 17033, USA; mfu5071@psu.edu; 2 Department of Pharmaceutical Sciences, Thomas J. Long School of Pharmacy, University of the Pacific, Stockton, CA 95211, USA

**Keywords:** SERCA pumps, calcium signaling, T cells, ER Ca^2+^ stores, T-cell activation

## Abstract

**Highlights:**

**What are the main findings?**

- Emerging evidence points to complex, specialized SERCA pump-regulated Ca^2+^ stores that can participate in producing and regulating the sustained Ca^2+^ signal required for T-cell activation;
- Underlying expanded novel roles of multiple SERCA-regulated T-cell Ca^2+^ stores is the increasing recognition that SERCA pumps operate in a microdomain milieu comprising an interactome controlling tailored SERCA functions.

**What are the implications of the main findings?**

- Knowledge of diverse and specific SERCA-regulated Ca^2+^ stores may enable productive T-cell engineering efforts yielding T-cell phenotypes with therapeutically desirable attributes for treatment of a range of immune system dysfunctional states;
- Better understanding of the SERCA microdomain milieu and binding partner interactions permits dissection of SERCA regulatory pathways and potential development of more comprehensively efficacious SERCA-targeted drug strategies.

**Abstract:**

T cells are the central regulators of the adaptive immune system, guiding both the cell-mediated and antibody-based elements of the immune response. Crucial to T-cell activation and differentiation, the T-cell receptor must transduce antigen exposure using a sustained elevated Ca^2+^ signal. A substantial body of research has identified and characterized multiple players in the Ca^2+^ signaling pathway, yet the sarcoplasmic/endoplasmic reticulum Ca^2+^-ATPase (SERCA) transporters, which intervene actively to regulate Ca^2+^ signal patterning and duration, remain relatively poorly characterized in the full scope of the T-cell signaling paradigm. In this review, we summarize the expanding research that is beginning to clarify the multiple complex roles SERCAs perform in shaping the information-rich Ca^2+^ signal. Pharmacologic modulators and other studies have revealed molecular and functional diversity in the SERCA pumps, with increasing recognition of their critical positioning in regulating ER Ca^2+^ store networks and functional roles, which ultimately derive from dynamic microdomain assemblies containing potentially highly tailored SERCA-binding protein interactomes. A better understanding of SERCA transporter functions underlies increasing interest in developing novel therapeutic strategies targeting these key ion pumps in efforts to engineer T-cell phenotypes for more therapeutically efficacious management of cancer, autoimmunity, and other immune-based pathologies.

## 1. Introduction

The T cell in the naïve state is selected by antigen stimulation to enter proliferation and differentiation cycles via engagement of a T-cell receptor (TCR) pathway that is heavily dependent on the generation of a Ca^2+^ signal [1,2]. Given the central role T cells play in coordinating the adaptive immune response, there is great interest in elucidating the details underlying TCR-activated Ca^2+^ signals. The current T-cell Ca^2+^ signaling paradigm describes the early antigen-induced signal propagating downstream via production of inositol 1,4,5-trisphosphate (IP3), acting in concert with a complex web of parallel protein kinases and other signaling mediators to push quiescent T cells into cell division and clonal expansion [3,4,5]. Not surprisingly, the TCR signaling system is acknowledged as a dauntingly complex aggregation of proteins linked to a well-recognized sequence of events, culminating in an IP3-induced Ca^2+^ signal essential for full T-cell activation. Current efforts continue to clarify the roles played by ion channels and other signaling mediators participating in T-cell Ca^2+^ signal generation and regulation [6]. In this review, we focus on the ATP-consuming ion-transport pumps of the sarcoplasmic/endoplasmic reticulum Ca^2+^-ATPase (SERCA) family as recent work is beginning to reveal an expanding role for these Ca^2+^ signal regulators in the full scope of T-cell functions, and thereby attracting interest as possible sites of therapeutic targeting for a suite of chronic poorly managed immune pathologies, including cancer and autoimmunity.

## 2. The T-Cell Ca^2+^ Signal Paradigm

A substantial body of research spanning more than thirty years has produced the well-accepted general view of how antigen stimulation recruits a TCR-activated Ca^2+^ signal that drives downstream nuclear factor of activated T cell (NFAT)-mediated gene expression events required to propel the cells into the proliferative phase [1,3,4,7,8]. The crucial first step of the pathway is the transduction of the antigen-bound TCR into sequential proximal tyrosine kinase activation, which is tightly coupled to IP3-mediated Ca^2+^ release from the endoplasmic reticulum (ER). There is also evidence that the TCR can produce an additional Ca^2+^-mobilizing messenger (nicotinic acid adenine dinucleotide—NAADP) acting on the less abundantly ER-expressed ryanodine receptor (RyR) in T cells [9,10].

**Figure 1:**
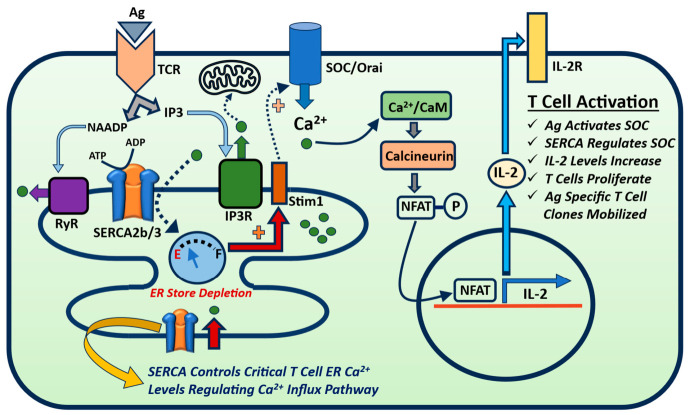
**The T-cell signaling paradigm.** The figure depicts the general pathway for T-cell activation and proliferation in response to antigen stimulation on the T-cell receptor (TCR). SERCA pumps are noted to play a key role in the T-cell activation paradigm by virtue of their active management of ER Ca^2+^ store levels. ER Ca^2+^ store depletion is the activation signal that induces store-operated Ca^2+^ (SOC) influx, which is essential to couple TCR activation into T-cell proliferation and clonal expansion. Abbreviations: Ag, antigen; ER, endoplasmic reticulum; SERCA, sarcoplasmic/endoplasmic reticulum Ca^2+^-ATPase; Stim1, stromal interaction molecule 1; NFAT, nuclear factor of activated T cells; IL-2, interleukin-2; IL-2R, interleukin-2 receptor; IP3R, inositol 1,4,5-trisphosphate receptor; RyR, ryanodine receptor; CaM, calmodulin; NAADP, nicotinic acid adenine dinucleotide phosphate.

In the T-cell signaling system, this initial IP3-mediated step appears primarily to serve as a type of “priming” event to produce an ER Ca^2+^ store depletion signal that activates Stim1 translocation and opening of the store-operated channel (SOC), Orai1, in the plasma membrane, enabling a secondary Ca^2+^ signal via Ca^2+^ influx (Figure 1). This view largely derives from subcellular characterization work that suggests that the ER organelle in T cells comprises a relatively small cytosolic volume, particularly compared to SR Ca^2+^ stores in excitable cells like skeletal and cardiac muscle [1,11,12]. Elevated cytoplasmic Ca^2+^, as a transient signal, is then rapidly captured and re-sequestered in the ER store by the SERCA pumps, with additional Ca^2+^ buffering contributed by mitochondrial Ca^2+^ uptake. The IP3-induced Ca^2+^ signal will activate ancillary and linked pathways (protein kinase C (PKC), calmodulin (CaM), etc.) participating in T-cell activation, but the depletion-generated signal perhaps carries greater significance in early T-cell activation given its essential role in engaging SOC-mediated Ca^2+^ influx [1,13,14,15]. Indeed, it is the sustained SOC-mediated Ca^2+^ influx response that has been clearly identified as the necessary signaling step that activates the protein phosphatase Calcineurin (CN), whose enzymatic action permits nuclear translocation and activation of NFAT with the subsequent expression of interleukin-2 (IL-2) and other growth-promoting genes in the T cell [4,13] (Figure 1). Thus, given the vital connection between the state of ER Ca^2+^ stores in coupling to the essential SOC-mediated T-cell activation pathway, the SERCA Ca^2+^ pumps continue to attract growing interest as a potentially much more complexly regulated target in the machinery of T-cell signaling.

## 3. Pharmacological Regulators Reveal Multiple T-Cell SERCA Profiles and Offer Clues into Disease States Linked to SERCA Dysfunction

The SERCA Ca^2+^ pumps operate according to the classic enzymatic sequence of the P-type ATP-driven ion pumps, cycling between E1 and E2 conformational states of alternating high/low Ca^2+^ affinity coupled to ATP hydrolysis [16,17,18]. Given their fundamental roles in cellular physiology, the SERCA pumps have been extensively characterized with respect to gene regulation and diversity, in addition to elucidation of their complex biophysical enzymatic mechanism. SERCA proteins are encoded by three separate genes (ATP2A1-3), which are subjected to alternative splicing events, producing multiple SERCA protein isoforms [19,20,21]. T cells express two main Ca^2+^-ATPase isoforms, SERCA 2b and SERCA 3, with further evidence revealing additional layers of protein diversity in the SERCA 3 gene family, accounting for as many as six SERCA 3 isoforms (SERCA 3a-f) [20,22,23]. Indeed, the extensive splicing actions and the isoform diversity suggest that T-cell SERCA function is likely subject to complex regulation that is perhaps linked to highly tailored T-cell signaling functions and elaborate specialized differentiation programs, which are required to produce multiple T-cell phenotypes deployed by immune response progression. Though currently unknown, it may be that different T-cell subsets spanning the helper, regulatory, and cytotoxic phenotypes utilize distinct SERCA 3 pump isoforms to confer unique Ca^2+^ signaling routines corresponding to their specialized functional roles in the immune system.

Decoding the complex antigen-induced intracellular signaling processes in T cells continues to be a daunting challenge in the domains of immunology and cell signaling research. Yet, a major contributor to our current understanding of the crucial T-cell Ca^2+^ signal has been the profitable application of a diverse group of structurally distinct, naturally occurring, and synthetic small-molecule SERCA pump modulators. Previous work, as well as ongoing research deploying SERCA pharmacology, reinforces the hypothesis that these critical Ca^2+^ solute transporters are complexly integrated within the dynamic and tightly regulated Ca^2+^ signal pathways initiated by TCR activation. The naturally occurring plant-derived sesquiterpene lactone compound thapsigargin (TG) is the consummate example of the productive use of SERCA pharmacology. For more than 35 years, TG has been recognized as a highly potent and uniquely specific inhibitor of SERCA pumps, contributing enormously over this time span to our understanding of ER Ca^2+^ stores and the obligately linked SOC Ca^2+^ influx pathway [24,25,26,27]. Indeed, early experiments using TG’s potent actions to deplete ER Ca^2+^ stores along with TCR-activated IP3-mediated Ca^2+^ release solidified the now well-accepted Calcium-Release Activated Current (CRAC) mechanism, containing the central Orai1 Ca^2+^-selective ion channel as the essential antigen-stimulated event underlying T-cell activation [28,29,30,31].

Given its very high potency, TG will block all SERCA isoforms; however, an intriguing action of TG’s effects has emerged, revealing that low doses of the compound can specifically inhibit the SERCA 2b pump isoform [23,32,33,34]. This TG-specific effect was initially described in platelets, but more recently has been documented and used in further characterization studies in T-cell populations [22,23,35,36]. The use of TG as a selective SERCA 2b blocker adds additional value to a compound with a long history of assisting our efforts to decipher T-cell intracellular Ca^2+^ signaling events. Thus, the basis of this approach is the use of TG at very low drug concentrations (picomolar levels), at which levels the SERCA 2b pump exhibits greater sensitivity to TG’s inhibitory actions than the SERCA 3 pump does. In the absence of advanced microscopic and other experimental approaches that enable unambiguous study of ER organelle substructure, utilizing specific modulatory actions of small-molecule compounds, like low-dose TG, as targeting agents on specific SERCA pumps provides a constructive path to gain insight into the multi-level ER Ca^2+^ store dynamics operating in the T-cell signaling milieu.

The specificity achievable via application of picomolar TG enables the targeted disruption of the SERCA 2b–regulated ER Ca^2+^ store, which is generally thought to be the main or largest pool of stored Ca^2+^ in T cells [37]. Indeed, according to the T-cell signaling paradigm mentioned above, it is the SERCA 2b–regulated Ca^2+^ pool that communicates the critical activation signal to the Orai1 Ca^2+^ channel subsequent to antigen-induced TCR stimulation and IP3 production driving cell proliferation. Thus, low-dose TG treatment enables scalable, specific perturbation of SERCA 2b Ca^2+^ stores, likely mimicking SERCA 2b dysfunction observed in disease states, and permitting unique insights into the diverse roles SERCA 2b pumps play in T-cell Ca^2+^ signal regulation. As such, the low-dose TG treatment regimen presents the opportunity to interrogate SERCA 2b defects that impair ER Ca^2+^ loading that would result in a “leakier” Ca^2+^ store; these are critical, hypothesized T-cell maladapted phenotypes expected to alter the key ER priming action on plasma membrane Orai1-mediated Ca^2+^ influx. Indeed, dysregulation of the high-affinity Ca^2+^-transporting SERCA 2b pumps, targeted by low-dose TG exposure, underlies the increasing suspicion that chronic ER Ca^2+^ depletion may account for the disrupted T-cell signaling defects observed in metabolic disorders and pathologic immunodeficiency (see below) [14,38,39,40,41,42].

**Figure 2:**
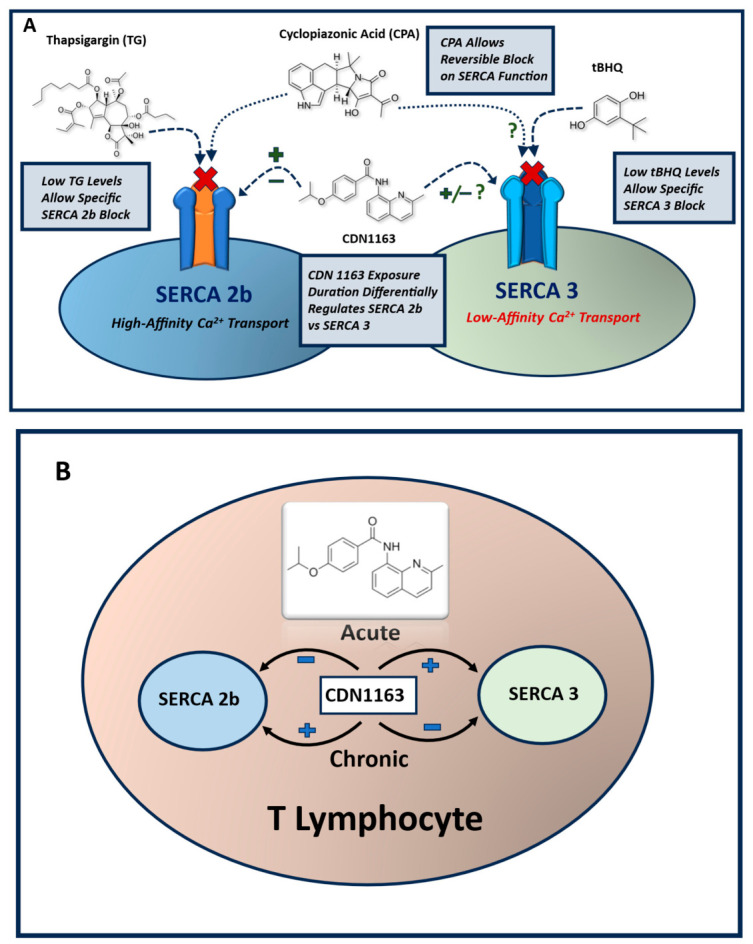
**Key group of structurally distinct pharmacological compounds that have contributed to a better understanding of SERCA functions in T-cell signaling pathways.** (**A**) The figure shows the main group of pharmacological agents that have contributed to providing additional insight into how the distinct SERCA pumps function in T-cell signaling physiology. The + and − symbols in the figure denote the modulatory actions of the drugs on the SERCA enzymes, with + indicating pump stimulation and − indicating pump inhibition. The particularly useful properties of the compounds, thapsigargin, cyclopiazonic acid, tBHQ, and CDN1163 are indicated. (**B**) Differential actions of CDN1163 on T-cell Ca^2+^ stores depicting time-dependent and SERCA isoform effects, implicating distinct roles of SERCA pumps in T-cell signaling functions. The + and − symbols in the figure denote the modulatory actions of the drugs on the SERCA enzymes, with + indicating pump stimulation and − indicating pump inhibition.

The SERCA pharmacological toolkit was further enhanced with the recognition that the synthetic SERCA modulator, 2,5-di-(*tert* butyl)-1,4-benzohydroquinone (tBHQ), at low concentrations could specifically inhibit the SERCA 3 pump isoform [22,23,32] (Figure 2). As with the low-dose TG discovery, tBHQ’s specific inhibitory action on SERCA 3 was originally and most fully characterized in platelets [32,33,43]; however, the same specific inhibitory effect has been reported in Jurkat and other lymphocyte populations [22,23,35]. The application of tBHQ to specifically perturb SERCA 3–regulated ER Ca^2+^ stores enables further characterization of a putative distinct T-cell Ca^2+^ pool, hypothesized as a unique SERCA 2b–companion regulator of intracellular Ca^2+^ signaling, as will be described below. Indeed, it has long been recognized that the SERCA 3 Ca^2+^ pump possesses distinct enzymatic differences from SERCA 2b transporters, most conspicuously in having a lower-affinity Ca^2+^ binding site [44,45]. This feature, of course, has led to the compelling idea that SERCA 3, unlike SERCA 2b, is likely to operate in a high intracellular Ca^2+^ domain, and thus could potentially represent a separate Ca^2+^ pool participating in a unique constellation of T-cell Ca^2+^ signal-regulated events.

Another prominent SERCA modulator that, like TG, has been used over the past several decades of research is the natural product compound cyclopiazonic acid (CPA) [46,47,48]. The distinctive property of CPA compared to TG and tBHQ is the reversibility of its inhibitory action on SERCA [11,12]. Indeed, TG is difficult to use from this perspective, given that it binds to the SERCA pumps with such high affinity that it cannot be easily removed; therefore, SERCA-regulated ER functions are permanently corrupted when cells are treated with the compound. There is some uncertainty as to the SERCA specificity of CPA, yet it appears, like low-dose TG, to exert some preferential blockade on the SERCA 2b Ca^2+^ pumps relative to SERCA 3 when applied in the low micromolar range [49]. CPA has been used in compelling experiments in Jurkat and primary human T cells to provide additional insight into the prevailing hypothesis that T-cell ER Ca^2+^ stores serve only a limited scope, acting merely as a priming signal for the dominant Ca^2+^ influx response in T-cell activation. CPA was used in these experiments to quantitatively analyze T-cell Ca^2+^ store capacities and to show that SOC channels mediate a Ca^2+^-induced Ca^2+^ release (CICR) mechanism in T cells via IP3Rs and RyRs, analogous to what has been observed in muscle cells [11,12]. Further, CICR via IP3R/RyR channel activation was shown to be essential for T-cell activation, acting in an indirect fashion as a negative regulator of SERCA activity by virtue of maintaining ER Ca^2+^ pools relatively depleted within the confines of a Stim1/Orai1 microdomain. The key findings of this seminal work suggest that CICR-based Ca^2+^ release from the ER keeps SOC channels from inactivating and contributes a significant fraction of the composite Ca^2+^ signal of T-cell activation, previously thought to be a minor contributor compared to SOC-mediated Ca^2+^ influx [11,12].

All of the foregoing small-molecule SERCA modulators are inhibitors of the Ca^2+^ pumps; thus, a deeper understanding of SERCA function must be attained through the use of compounds with a SERCA-activating action, serving as an effective complementary pharmacology toolset that enables both downregulation as well as upregulation of SERCA activity. Given its complex enzymatic and biophysical properties, it is considerably more challenging to identify a SERCA pump activator, yet a highly versatile and productive small molecule did emerge from high-throughput screening of a 20,000-compound library—a drug ultimately designated as CDN1163 [50]. CDN1163 was originally identified in assays designed to spot compounds that could activate cardiac SERCA 2a via disruption of its interaction with the native cardiac SR-resident SERCA inhibitor phospholamban (PLB) [50]. It was discovered, however, that CDN1163 could also activate the skeletal muscle SERCA 1a pump isoform, albeit with differing efficacy, yet nevertheless showing that the compound could activate SERCA pumps more broadly. Indeed, several studies have utilized CDN1163 in experiments that have revealed a range of salutary effects of stimulating SERCA pumps in a variety of cell- and tissue-specific contexts, with most dealing with cardiac and/or skeletal muscle systems [51,52,53,54,55].

More recent experiments using CDN1163 in nonmuscle cells have revealed more complex effects of the compound on SERCA-regulated ER Ca^2+^ stores [35,56,57]. In Jurkat T cells, for example, CDN1163—like the SERCA blockers described above—was observed to exert complex time-dependent effects with apparent opposing alterations on SERCA 2b and SERCA 3 Ca^2+^ pumps [35] (Figure 2A,B). Indeed, using the low-dose TG/tBHQ regimen described above, experiments revealed that CDN1163 appeared to be inducing a “leaky” state of the SERCA 2b–regulated Ca^2+^ pools within the first few minutes of exposure in T cells, acting as though the compound was inhibiting SERCA 2b. This effect has also been reported in neurons and lung epithelial cells that also contain SERCA 2b–regulated ER Ca^2+^ stores [56,57]. Intriguingly, CDN1163 treatment induced the opposite effect on SERCA 3–regulated Ca^2+^ pools in T cells, revealing upon early exposure an augmentation of Ca^2+^ release from these stores as though the compound was indeed acting as a SERCA activator. Yet, a paradoxical time-dependent profile was subsequently observed in T cells, whereby with extended CDN1163 exposure (≥12 h), ER Ca^2+^ loading into the SERCA 2b–regulated Ca^2+^ pools was enhanced, while the reciprocal experiments revealed a diminished Ca^2+^ release response from SERCA 3–regulated pools, in accordance with the asymmetric effects of CDN1163 noted in the short-term experiments [35]. Thus, these experiments suggest an intriguing differential mode of CDN1163-mediated SERCA regulation in T cells, with acute exposure alternately inhibiting SERCA 2b function while stimulating SERCA 3 activity; yet, with prolonged or chronic exposure, these pharmacological effects appear to be reversed (Figure 2B). However, it should be emphasized that the gains from the application of SERCA pharmacology described above are necessarily limited due to unavoidable inherent nonspecific complex actions of small-molecule drugs, and thus can provide only an approximate view of SERCA pump expression patterns and function in the dynamic ER organelle environment (Figure 2A,B). Table 1 summarizes the major properties and T-cell effects of the SERCA pump modulators discussed in this review.

## 4. Diverse Roles of SERCA-Regulated T-Cell Ca^2+^ Stores Reveal Both Novel Signaling Functions and Perturbations of Immune System Disease States

**Figure 3:**
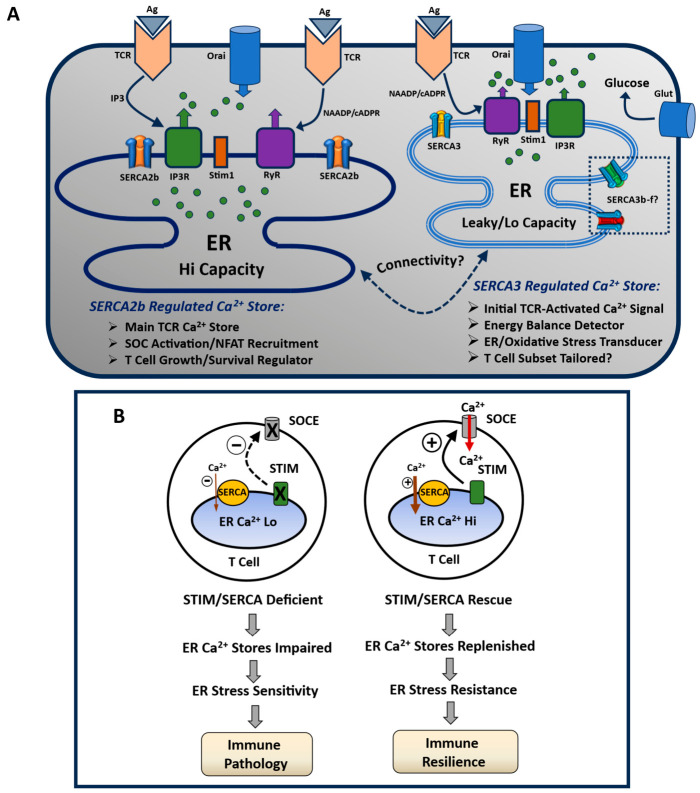
**Unique SERCA expression profile confers specific signaling functions on T-cell ER Ca^2+^ stores with increasing recognition of SERCA-based Ca^2+^ store defects in immune system disease states.** (**A**) Schematic representation of distinct ER Ca^2+^ stores in T-cell signaling landscape. The figure conveys size- and position-specific properties of the putative SERCA 2b– and SERCA 3–regulated ER Ca^2+^ pools. Also indicated are brief summaries of the general signaling and physiological functions attributed to specific SERCA isoforms, which confer unique functional properties to ER Ca^2+^ stores. (**B**) The figure highlights the strategic role of SERCA function, with its coupled action to regulate store-operated Ca^2+^ entry (SOCE) as a paramount feature of robust T-cell function. The figure underscores a major motivating strategy to target SERCA pump function as a means to endow T cells with functional resilience to counter T-cell exhaustion with better tumor-eradicating efficacy. Abbreviations: SOCE, store-operated Ca^2+^ entry; ER, endoplasmic reticulum; SERCA, sarcoplasmic/endoplasmic reticulum Ca^2+^-ATPase; Stim, stromal interaction molecule; Glut, glucose transporter; IP3R, inositol 1,4,5-trisphosphate receptor; RyR, ryanodine receptor; Ag, antigen; TCR, T-cell receptor.

The SERCA pump pharmacological modulators described above, along with other studies, have begun to expand the canonical T-cell signaling paradigm to suggest the presence of a more complex network of potentially multiple intracellular SERCA-regulated Ca^2+^ stores performing more specialized or tailored roles in regulating T-cell Ca^2+^ signal pathways (Figure 3A). Certainly, the low-dose TG/tBHQ- and CDN1163-focused studies suggest that SERCA 2b and SERCA 3 are expressed on distinct ER subcompartments, with putatively distinct functions in the T-cell signaling landscape [35]; yet, it remains entirely plausible that there could exist either continuous or discontinuous connectivity between a largely SERCA 2b–regulated and companion, smaller SERCA 3–regulated ER Ca^2+^ storage site.

Indeed, SERCA 3–regulated ER Ca^2+^ stores may occupy cytosolic microdomain-delimited regions of elevated local Ca^2+^ concentration levels given the lower Ca^2+^ affinity of this pump compared to SERCA 2b. Platelet studies have revealed that SERCA 3 transporters appear to be expressed on distinct ER pools that exhibit a high native Ca^2+^ permeability or “leakiness”, consistent with regulation by a SERCA enzyme with lower Ca^2+^ affinity than ER stores expressing the SERCA 2b pump [58,59,60]. The SERCA 3 ER stores in platelets were also shown to be responsive to a unique subset of Ca^2+^-mobilizing agents, further implying distinct, and perhaps physically separated, ER organellar compartments [33,61]. Moreover, an intriguing overlapping spectrum of Ca^2+^-releasing ER store functions was observed in platelet signaling; it was noted, for example, that ryanodine-sensitive ER pools were affiliated specifically with Ca^2+^ stores regulated by SERCA 3 pumps, whereas the platelet activator thrombin induced Ca^2+^ release from both SERCA 3– and SERCA 2b–regulated ER stores [62]. A similar Ca^2+^ release profile was recently observed in T cells, revealing that NAADP mobilized Ca^2+^ from RyR-sensitive pools expressing the SERCA 3 pump isoform, while thrombin-mediated Ca^2+^ release appeared to be linked to both SERCA 2b– and SERCA 3–regulated pools [35]. Intriguingly, in platelets, thrombin was shown to initially stimulate a rapid NAADP/RyR-mediated Ca^2+^ signal from SERCA 3–regulated stores, with an initial small ADP secretion response; this initial ADP response, however, was further amplified secondarily by thrombin’s actions to stimulate IP3 production and Ca^2+^ release from SERCA 2b–regulated pools [62]. Thus, a single agonist was observed to produce both IP3- and RyR-mediated Ca^2+^ signals by accessing Ca^2+^ stores regulated separately by SERCA 2b and SERCA 3. Notably, antigen-activated TCRs also represent a receptor system that recruits both IP3- and RyR-mediated signaling responses as well [10,12,63]. These thrombin signaling actions in platelets further underscore how SERCA 2b– and SERCA 3–regulated Ca^2+^ stores may operate in concert to produce patterned signaling outcomes in platelets and T cells, validating the necessity of multiple SERCA isoforms in Ca^2+^ signaling dynamics.

In T cells, as in most other cells, the SERCA 2b–regulated ER Ca^2+^ stores appear to be higher-capacity Ca^2+^ pools compared to stores regulated by the SERCA 3 pumps [37,64]. The SERCA 2b Ca^2+^ stores are, therefore, more likely integrated into the protracted multi-hour elevated cytosolic Ca^2+^ signal required for full T-cell activation. As such, the SERCA 2b–regulated ER appears to participate extensively in T-cell growth regulation, with earlier studies revealing an increase in SERCA 2b expression levels in comparison to SERCA 3 protein levels during T-cell activation and proliferation [65]. Recent experiments using the SERCA activator CDN1163 in combination with the low-dose TG/tBHQ treatment protocol have also revealed improved support for T-cell survival and growth mediated by enhanced SERCA 2b activity [35].

Moreover, as previously noted, CDN1163’s actions to preferentially activate SERCA 2b in T cells were exploited to show that the augmented SERCA 2b function contributed to improved ER Ca^2+^ loading and T-cell viability in response to chronic diabetic hyperglycemic stress [38]. Indeed, this salutary effect of CDN1163 on SERCA-regulated Ca^2+^ ER stores has been amply demonstrated in settings of metabolic and cardiovascular pathologies, and now, recent findings suggest this may be a specific effect of targeting the SERCA 2b pump in T cells [52,55,66]. These experiments motivate interest in developing targeted therapies to modulate SERCA 2b functions, which increasingly appear to underlie critical metabolic defects that impair T-cell functions associated with immune system dysfunction. Notably, this phenotype was indeed observed in reports revealing reduced lymphocyte SERCA 2b expression levels and depleted ER Ca^2+^ stores using a prominent rat model of Type 2 Diabetes, with implications of a metabolic disorder likely underlying defective T-cell functions linked to well-recognized immune function impairment in the diabetic state [38].

Thus, a T-cell axis of SERCA 2b/SERCA 3 differential functions appears to be steadily emerging in the T-cell signaling regime. Evidence points to an intriguing temporal order of Ca^2+^ release/influx events linked to selective TCR signals acting on distinct ER Ca^2+^ pools, likely expressing distinct SERCA pump isoforms. This view postulates that the RyR activator NAADP is the first TCR-induced signal initiating Ca^2+^ release and SOC-mediated Ca^2+^ influx within the first few seconds of TCR engagement, followed a few seconds later by a more global amplification and propagation of sustained Ca^2+^ influx activity due to both RyR and IP3R activity [67,68]. Curiously, the initial RyR-mediated Ca^2+^ signal appears to arise from a unique topography of “pre-configured” ER Ca^2+^ pools exhibiting resting-state contacts between Orai1-plasma-membrane localized channels and underlying ER-localized RyR and Stim1 channel regulators, reminiscent of excitation–contraction coupling systems in muscle [63,68,69] (Figure 3A). Indeed, this pre-configured ER Ca^2+^ pool, recruited first in the TCR pathway, may be a store selectively regulated by SERCA 3 Ca^2+^ pumps given the observation, mentioned above, that RyRs appear to be expressed predominantly in SERCA 3–regulated ER stores in both platelets and T cells. It is interesting to note that, in platelets, which share a similar SERCA 2b/SERCA 3 functional dichotomy with T cells, this SERCA 3–regulated ER Ca^2+^ store is less sensitive to cytoskeletal disruption compared to SERCA 2b–regulated ER Ca^2+^ stores in their trafficking migration to plasma membrane SOC channels; this is consistent, as noted above, with the SERCA 3–regulated ER store’s positioning, which is in close contact with the plasma membrane [64] (Figure 3A).

In addition to its topographical plasma membrane–localized domain and distinct Ca^2+^ signaling sequence and agonist sensitivities, SERCA 3 functions also appear to be increasingly distinguishable from SERCA 2b functions relating to T-cell management of the ER stress condition. T cells, in many respects, are a uniquely vulnerable cellular phenotype to the ER stress state, given the demanding energy requirements of the complex signaling milieu needed to sustain active proliferation/differentiation programs along with their extensive migratory and trafficking proclivity. In this context, it is interesting to note recent reports that demonstrate an intriguing interconnectivity between expression levels of the two major pump isoforms, revealing an increase in SERCA 2b levels accompanied by a corresponding decrease in SERCA 3 levels as cells undergo de-differentiation and proliferation [65,70,71]. Similarly, recent investigations have suggested that SERCA 3 expression levels appear to be more sensitively coupled to key ER/oxidative stress inputs, such as glucose uptake and antioxidant production [36]. Perhaps the uniquely “leakier” state of SERCA 3–regulated ER, along with its lower Ca^2+^ affinity, confers a more specialized energy sensor–property coupling, falling ATP levels to a quantitatively greater ER Ca^2+^ store depletion than is attainable in the higher-affinity SERCA 2b–regulated pools. Thus, in this putative mechanism, lower-affinity Ca^2+^ SERCA 3 stores would accommodate greater Ca^2+^ depletion due to reduced ATP levels, and thereby serve as an “energy” stress–sensor coupling to pathways that increase glucose uptake and utilization. Indeed, specialized SERCA 3 functions may ultimately be identified as “tunable” parameters to subserve specialized needs within the range of complex T-cell phenotypes. Certainly, the scope of T-cell differentiation pathways is considerable, with an increasing array of specialized T-cell functions residing in helper, cytotoxic, and regulatory subsets. Moreover, we are perhaps provided with a compelling hint that T cells utilize specialized SERCA 3 Ca^2+^ pump functions tailored to unique signaling demands, given that no less than three distinct SERCA 3 pump isoforms have been identified in T cells [20,45].

As mentioned previously, T cells confront a multitude of challenging, potentially maladaptive environments with severe energy constraints and high levels of oxidative stress, navigating chronic inflammatory conditions associated with autoimmunity and cancer. Many previous investigations have reported on SERCA 2b functional alterations in metabolic and cardiovascular disorders, highlighting the possibilities of designing rational therapeutic strategies for targeting SERCA 2b [51,52,72,73]. However, in T-cell biology, it has been less clear if SERCA isoform functional diversity and impairment are associated with unambiguous T-cell and immune-response dysfunction. Yet, this pattern is clearly changing, with new observations linking SERCA functions to T-cell immunodeficiency and cell proliferation cycles. In addition, intriguingly, a recent study reported that SERCA deficiency impaired VDJ antigen-receptor recombination in B-cell development, a developmental process also known to occur in T-cell development in the thymus and likely linked to severe immunodeficiency [40,74,75,76].

As alluded to above, it was recently reported that chronic hyperglycemia induced a steady, protracted decline in SERCA 2b expression levels and a concomitant loss in ER Ca^2+^ storage levels [38]. Moreover, it was also observed in these studies that early in the course of hyperglycemia, SERCA 3 and Stim1 protein levels increased even while SERCA 2b levels were declining. Thus, it appears, as mentioned above, that SERCA 3—along with other mediators such as Stim1—may represent a more quickly mobilized adaptive response to balance metabolic and energy perturbations in T cells subjected to hyperglycemic stress. Indeed, these are the types of maladaptive environments increasingly thought to underlie changes in T cells that lead to the appearance of the T-cell exhaustion phenotype, permitting tumor escape from immune eradication [77,78,79,80,81,82]. Thus, recent efforts have been directed to finding ways to endow chimeric antigen receptor (CAR) T cells with greater metabolic and functional resilience in the tumor microenvironment [78,80,82]. A recent study, for example, noted a much-improved CAR T-cell anti-tumor responsivity in T cells engineered to overexpress the major T-cell glucose transporter Glut1, achieving both robust energy utilization as well as increased oxidative stress resistance [83]. As noted above, the SERCA 3 Ca^2+^ pump may also be a productive target for improved CAR T-cell therapy, given its apparent capacity to confer both increased antioxidant production as well as Glut1-mediated glucose uptake. Thus, strategic approaches to augment SERCA 2b and/or SERCA 3 functions to achieve robust Ca^2+^ store functions and energy utilization could be productively adopted to generate an improved engineered T cell with suitable resilience and functional efficacy in inhospitable inflammatory and tumor microenvironments (Figure 3B). Certainly, however, a much more detailed understanding of specific SERCA pump isoform functions would be needed, as a single universally engineered SERCA-modulated T-cell phenotype would not suffice for therapeutic application of the full spectrum of immune system pathologies. It may, for example, be possible to produce a SERCA 3–modulated cytotoxic T-cell phenotype to confer metabolic resilience and durability for effective anti-tumor responses, while conversely targeting SERCA 2b–modulated T-cell functions to abrogate activation pathways tending to produce hyper-stimulated T-cell responses associated with autoimmune conditions. Similarly, future T-cell engineering efforts may be able to expand and stabilize critical regulatory T-cell subsets via specific SERCA pump modulation to curtail chronic hyper-inflammatory autoimmune states.

Table 2 provides a brief summary of our current understanding of properties and T-cell functional roles of the major SERCA pump isoforms discussed in this review. As previously mentioned, although multiple SERCA 3 pump isoforms (SERCA 3b-3f) have been detected in T cells, there continues to be uncertainty as to the expression and specific functions of these novel isoforms in differentiated T-cell subsets (Table 2).

## 5. Spectrum of Potential Binding Partners Suggest Multiple Regulatory Levels of the T-Cell SERCA Pumps

**Figure 4:**
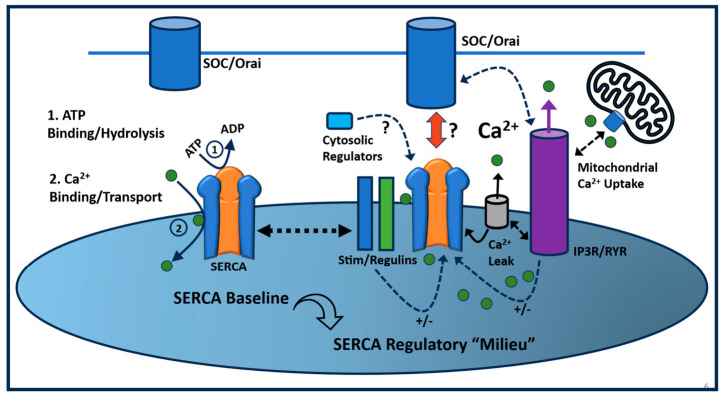
**Hypothetical SERCA interactome represents potential assembly of functionally tailored microdomain of SERCA-regulating binding partners**. The figure depicts a range of known and putative regulators acting on the baseline SERCA function of enzymatic ATP hydrolysis and Ca^2+^ transport. A rich potential of SERCA-binding components lies within the ER membrane, cytosol, and plasma membrane, thought to be able to assemble a de novo regulatory milieu and thereby considerably expand specialized and tailored signaling scaffolds to drive differentiated T-cell functions. The + and − symbols in the figure denote the modulatory actions of putative protein regulators on the SERCA enzymes, with + indicating pump stimulation and − indicating pump inhibition. Abbreviations: SERCA, sarcoplasmic/endoplasmic reticulum Ca^2+^-ATPase; SOC, store-operated Ca^2+^ channel; IP3R, inositol 1,4,5-trisphosphate receptor; RyR, ryanodine receptor; Stim, stromal interaction molecule.

The preceding sections describe a rich and extensive SERCA pharmacology, as well as growing recognition that T-cell SERCAs are likely to participate in selective and specialized roles in the complex spatiotemporal Ca^2+^ signaling landscape of T cells. These expanded, novel roles of the SERCA pumps, nevertheless, will ultimately be based on the collective, and likely rich, tapestry of molecular interactions of regulatory factors acting on SERCAs in the ER membrane domain. Certainly, as a major Ca^2+^-sequestering transporter, the SERCA pumps occupy a particularly pivotal role given that they control key levels of excitatory cytosolic Ca^2+^ signals but also preside over keeping ER Ca^2+^ levels within a manageable range to avoid triggering the deleterious ER stress pathway [45,51]. Thus, it is not surprising that SERCA pumps would have evolved a complex “SERCA regulatory milieu” of interactions with the full spectrum—ER-resident, PM-resident, and/or cytosolic-resident—of potential molecular regulators to manage cytosolic Ca^2+^ signal patterning as well as major facets of ER organelle integrity (Figure 4).

Notably, we owe much of our current understanding of SERCA regulation to the multi-decade research efforts examining SERCA systems in skeletal and cardiac muscle [84,85]. The abundance of SR membrane and the high expression levels of SR proteins have provided a tissue platform to investigate SERCA enzymatic and Ca^2+^ transport activity more easily [86,87]. These early efforts identified the most unambiguous SERCA regulator in characterizing the actions of cardiac SR phospholamban (PLB) as a premier SERCA modulator—an SR-localized peptide SERCA 2a inhibitor that still serves as a useful paradigmatic model of how a signaling pathway couples to modulation of SERCA activity [88,89,90,91,92]. Additional members of the “regulin” family of PLB-like SERCA modulators have been identified, including sarcolipin, myoregulin, and a class of “Another Regulin” (ALN) ER-localized peptides acting as SERCA regulators thought to be involved in thermogenesis [93,94,95,96,97]. With the exception of PLB, the regulin group of SR/ER peptides signify the challenge of SERCA regulation in that many of these peptide regulators are characterized by low expression levels and inconsistent representation in SR or ER membranes, particularly in nonmuscle tissues. Yet, like PLB, these SERCA modulators manifest the key concept that real-time dynamic alteration of the baseline SERCA enzymatic functions of ATP hydrolysis with coupled Ca^2+^ transport can be rapidly recruited to shape complex Ca^2+^ signal patterns in space and time, as well as to govern the integral ER store depletion/repletion cycles embedded in a myriad of T-cell functions (Figure 4).

Currently, the PLB and regulin family of peptides appear to be the dominant theme whereby an SR-/ER-resident protein juxtaposed to SERCA pumps can exert a regulatory influence on the enzymatic and Ca^2+^ transport actions of the pumps, with most acting as SERCA inhibitors. Intriguingly, a heterogeneous group of other putative SERCA regulators, distinct from the SR/ER peptide theme, have been described, albeit with a fairly limited number of studies to support these novel modes of SERCA regulation. The presenilins, for example—as potential culprits in Alzheimer’s disease pathology—have been described as ER-localized activators of the SERCA 2b pump isoform, and, thus, have been proposed as potential drug targets for amelioration of the Alzheimer’s neurodegenerative condition [98,99]. In contrast to SERCA regulators residing in the ER membrane, a group of cytosolic agents have been reported to exert regulatory effects on the SERCA pumps. The protein Sorcin, for example, which like presenilin is implicated in Alzheimer’s disease, has been reported to increase Ca^2+^ uptake in neurons via activation of the SERCA 2b pump isoform via its binding action as a cytosolic regulator [100]. Similarly, in T cells, the diverse group of protein immunophilins—with multiple diverse actions in lymphocyte biology—have been described to be SERCA modulators, with the cytosolic cyclophilin A species observed to act specifically in stimulating SERCA 2b activity [101]. Additionally, as alluded to above, T cells exhibit high energy demand to support the prolonged, elevated Ca^2+^ signaling needed for cell activation and proliferation. Thus, a recent study reported a novel mode of SERCA regulation to accommodate the protracted phase of elevated Ca^2+^ signals, in which the glycolysis intermediate phosphoenolpyruvate (PEP) acts as a unique SERCA 2b pump inhibitor [102]. As T cells are consuming glucose for energy support, PEP is generated via the glycolytic pathway and exerts an inhibitory effect on SERCA 2b, thereby maintaining elevated cytosolic Ca^2+^ levels to stimulate NFAT activity and cell proliferation.

Indeed, arguably the most compelling mode of SERCA regulation likely operating in a T-cell signaling landscape is not mediated by a constitutive mechanism, but rather by a de novo assembly of a directly and/or indirectly acting suite of SERCA-binding partners in spatially delimited domains of closely apposed ER and PM locales, representing the SERCA “regulatory milieu” (Figure 4). Yet, this type of SERCA pump regulation is particularly challenging to decipher given the difficulty of capturing transient interactions of proteins, which are often expressed at low levels in dynamically grouped spatiotemporal configurations conformed by the rapidly changing state of the ER Ca^2+^ stores. Notwithstanding the technical challenges, there is a growing body of experimental evidence consistent with the formation of a signal-induced ER/PM scaffold of interacting mediators directly or indirectly modulating SERCA activity [11,12,103,104,105,106]. As above, referring to the useful platelet system, experiments revealed that ER Ca^2+^ store depletion led to the assembly of an intracellular signaling microdomain comprising PM SOC channels in physical contact with ER-resident Stim1, IP3Rs, and SERCA 3 pumps [107,108,109,110]. The logic underlying this grouping of proteins is clear: IP3Rs mediate Ca^2+^ release, effecting ER store depletion that then physically couples to SOC channels to activate Ca^2+^ influx with the apparatus, and then activating SERCA 3 pumps to replenish ER Ca^2+^ stores on a timescale that prevents induction of oxidative and ER stress pathways. In this system, multiple possible SERCA-binding sites could exist within the protein milieu of the microdomain, yet the main SERCA regulator appears to be Stim1, which was shown to physically associate with the SERCA pump to increase Ca^2+^ uptake, efficiently coupling its role as an ER Ca^2+^ sensor to modulatory actions on the major solute transporter controlling ER Ca^2+^ levels [108].

In addition, as mentioned previously, these types of intracellular spatial microdomains have also been detected in T cells, revealing the pre-stimulus cluster of specialized SERCA-expressing ER membranes engaged with PM SOC channels via direct physical contacts with “trigger-ready” RyRs and Stim1 mediators awaiting TCR activation [63,68,69,111]. It might be anticipated, as in platelets, that Stim proteins or other “packaged” components of these assembled structures will regulate SERCA function to ensure that ER Ca^2+^ store balance is maintained for the duration of the specific antigen-activated T-cell response. Indeed, the intricate signaling specificity governing elaborate T-cell activation and differentiation pathways may lie in the diverse scope attainable from the rapid assembly of these Ca^2+^ signaling units that will confer a unique signaling spatiotemporal footprint based on the adaptable and readily exchangeable protein constituents contained in the scaffold. Thus, it was recently shown that the putative SERCA regulator Sorcin demonstrated this type of multi-targeted action by both inhibiting RyR channel activation while also directly stimulating SERCA 2b–mediated ER Ca^2+^ uptake [100].

The constellation of potential SERCA regulatory proteins operating in a T-cell Ca^2+^ signaling microdomain has been growing over the past several years of investigation. As mentioned previously, a central functional tenet underlying the ER Ca^2+^ store “interactome” is the ER Ca^2+^ leak pathway, a natively and enigmatically high resting permeability of the ER membrane to Ca^2+^ flow, with its apparent strategic linkage to energy metabolism. Not surprisingly, the scale of the ER Ca^2+^ leak is expected to be coupled to SERCA functional activity responding to a conditional signal, attributable both to ER Ca^2+^ store depletion as well as a potential state of ER Ca^2+^ store hyper-repletion. Although the molecular identity of the ER Ca^2+^ leak pathway remains elusive, the bcl-2 family of apoptosis regulators has garnered some advocacy as candidate mediators, with some experimental evidence also revealing the capacity to interact with and regulate SERCA activity [51,112]. In this context, SERCA pumps contained within an assembled microdomain manage the dynamic ER Ca^2+^ load by counteracting an overactive leak response, which depletes stores, along with the possible expanded role of interacting with IP3Rs to “off-load” excessive ER Ca^2+^ levels via IP3R-mediated transfer to mitochondria, which can also serve as a cytosolic Ca^2+^ buffer (Figure 4).

An earlier seminal study describing the actions of the Partner of Stim1 (POST) protein has offered a useful template for current studies on Ca^2+^ signal microdomains, underlining the scale of potential interacting regulatory proteins in the de novo assembly of these functional signaling units [113]. Indeed, in this work, the POST protein was revealed to physically interact with a broad range of ion-signaling partners, including SERCA, Stim1, plasma membrane Ca^2+^-ATPases, Na^+^/K^+^-ATPase, and SOC (Orai1) channels. Crucially, this scaffolding action of the POST protein, with its multiple contacts to other ion signaling mediators, was induced by the depletion of ER Ca^2+^ stores in T cells, lending experimental support to the plausibility of rapid assembly of a complex network or signaling platform mobilized by the critical ER Ca^2+^ depletion signal [113].

Intriguingly, SERCA pharmacology, as mentioned above, produces T-cell signaling effects that also appear to be consistent with the assembly of a multi-component Ca^2+^ signal microdomain apparatus. The disparate effects, for example, of CDN1163 as a putative SERCA activator, along with the older Ca^2+^ signal modulator 2-aminoethoxydiphenyl borate (2-APB), are arguably best described by drug actions on a composite site of multiple proteins assembled as a functional microdomain structure [35,114,115,116]. Thus, when CDN1163 is first added to T cells, it somewhat paradoxically, for a putative SERCA activator, induces an ER Ca^2+^ leak response. However, when the cells are exposed to the drug for longer time intervals, a more clearly acting SERCA stimulation manifests, with greater ER Ca^2+^ store loading and improved resilience to ER stress and growth inhibition. A plausible explanation, therefore, of CDN1163’s actions is a time-dependent sequela of events within an adaptable protein milieu that transforms the ER microdomain into a functional unit that accommodates SERCA activation, similar to that described above by Stim1’s actions, to stimulate SERCA activity in platelets. Furthermore, this delayed SERCA stimulatory action in T cells was observed to be specific for the SERCA 2b pump isoform, with a much different effect noted on SERCA 3, which additionally argues that the SERCA interactome within the domain of these delimited ER subregions can comprise a distinct profile of constituent mediators such that the drug produces a much different response dependent upon its unique environment. In a similar vein, the older drug 2-APB was associated with complex effects thought to be due to a generally nonspecific mode of action, as it was variously described first as an IP3R blocker and, subsequently, as both an activator and inhibitor of SOC activity as well as a SERCA inhibitor. Indeed, the unique sensitivity of these particular proteins (IP3R, SOC, and SERCA) to different concentrations of 2-APB was subsequently interpreted as a likely clue that the drug could be producing these ostensibly distinct effects simply by regulating composite protein interactions in distinct assembled microdomain units. Thus, depending on the particular milieu, 2-APB at certain concentrations was observed to stimulate SOC activity along with inhibiting SERCA pump activity; meanwhile, in other environments or at different drug levels, 2-APB was observed to inhibit SOC activity and block IP3R functions.

## 6. Conclusions

The role of the SERCA pumps has been gradually evolving from an initial, relatively limited, homeostatic role in the contraction/relaxation cycle of muscle physiology to a complexly active site integrated as a critical node in shaping and regulating Ca^2+^ signals. The recognition that a particular pump isoform, SERCA 2a, in cardiac muscle was subject to regulatory control by the SR-localized PLB protein set in motion the idea that these crucial ion-transporting enzymes could be embedded in signaling systems where they play essential roles in producing information-rich spatiotemporal Ca^2+^ signals. This view, which now accepts an expanded signaling-aligned function of the SERCA family of Ca^2+^ pumps, naturally leads to the hypothesis that the T cell likely utilizes SERCAs in diverse signaling programs, most convincingly because the cell not only expresses the “housekeeping” SERCA 2b pump, but also perhaps as many as six different SERCA 3 pump isoforms (SERCA 3a-f). Indeed, the molecular diversity of SERCA pump potential in T lymphocytes may functionally endow T-cell phenotypes with unique metabolic and signaling features encoding an impressive range of cellular functions, which is not surprising given the extensive diversity increasingly recognized in specialized T-cell subsets. We do emphasize, however, that this SERCA pump “potential” appears to be confined to the SERCA 3 gene family in T cells, and that while it is an interesting conjecture for SERCA 3 isoform diversity, there currently is very little direct evidence that distinct pump proteins function uniquely in different T-cell subsets.

Current research continually strengthens the hypothesis that SERCA transporters perform more specialized functions in adapting TCR Ca^2+^ signaling pathways to suit an increasingly rich and complex array of specialized T-cell functional outputs. In addition to influencing real-time Ca^2+^ signaling patterns, SERCA pumps are charged with the important role of maintaining ER Ca^2+^ store integrity by re-sequestering ER Ca^2+^ rundown due to signal discharge or combating depletion secondary to ATP consumption and resting Ca^2+^ leakage—all of which, if not regulated, can precipitate damaging pathophysiological states and varying states of T-cell dysfunction. The availability and productive use of a unique set of pharmacological SERCA modulators have also moved the field forward, providing key insights into T-cell physiology. Thus, the strategic use of a complementary set of SERCA blockers and activators suggests novel roles of spatially and functionally distinct ER Ca^2+^ pools that accord with the multi-dimensional cycles built into the demanding and protracted Ca^2+^ signal elicited by TCR activation. Yet, as mentioned previously, the use of SERCA-targeted drugs is a methodological limitation, presenting only limited capacity to ascertain specific subcellular localization and functions of discrete SERCA pump isoforms in the dynamic and sustained TCR-activated Ca^2+^ signal. Experimental approaches that enable greater precision in specifically tracking SERCA isoform expression and ER sub-compartment association will be needed to clarify the complex roles of SERCA-regulated Ca^2+^ stores in T-cell functions more unambiguously.

SERCA pharmacology and other studies are also helping to decode T-cell SERCA functions at the most fundamental levels, including gaining better resolution on the interacting protein binding partners that comprise the coalescence of specialized signaling microdomains. Future experiments taking advantage of the increasing sophistication of powerful microscopic techniques, coupled with proteomic approaches, will likely begin to elucidate the multiple levels of SERCA engagement in the T-cell Ca^2+^ signaling regime. Thus, SERCA pumps are likely to increasingly become attractive targets for therapeutic intervention and also as possible key sites to explore in the fast-moving field of T-cell engineering to produce T-cell phenotypes with favorable attributes for treating cancer and autoimmunity.

## Figures and Tables

**Table 1:** Comparison of SERCA pharmacologic regulators with respect to inhibition/activation mechanism, selectivity, reversibility, and T-cell effects.

Drug	Mechanism	Selectivity	Reversibility	T-Cell Actions
TG	Blocks E2-E1 turnover	Low TG blocks SERCA 2b	No	Release SERCA 2b Ca^2+^ stores
tBHQ	Blocks E2-E1 turnover	Low tBHQ blocks SERCA 3	No	Release SERCA 3 Ca^2+^ stores
CPA	Reduces ATP binding affinity	SERCA 2b > 3	Yes	Depletion/repletion studies
CDN1163	Increase Vmax; activator	SERCA 2b > 3	Yes	Increase SERCA 2b activity relative to SERCA 3

**Table 2:** Comparison of major T-cell SERCA isoforms with respect to Ca^2+^ affinity, organelle location, T-cell expression patterns, and putative T-cell functions.

Isoform	Ca^2+^ Affinity	Ca^2+^ Store	T-Cell Expression	T-Cell Functions
SERCA 2b	High	Main ER store	High: General SERCA 2b > 3	Multiple; Growth Control
SERCA 3	Low	Main/sub ER store	Low: Selective SERCA 3 < 2b	Energy/Stress? High Ca^2+^ Domains
SERCA 3b-3f	Low	Sub ER?	Low: Variable?	?

## Data Availability

No new data were created or analyzed in this study.
